# Complete genome sequence of the widely used coaggregation fusobacterial strain PK1594

**DOI:** 10.1128/mra.01359-25

**Published:** 2026-02-12

**Authors:** Bibek G C, Shiqi Xu, Chenggang Wu

**Affiliations:** 1 Department of Microbiology & Molecular Genetics, the University of Texas Health Science Center 12340, Houston, Texas, USA; Portland State University, Portland, Oregon, USA

**Keywords:** *Fusobacterium nucleatum*, *Fusobacterium hwasookii*, PK1594, bacterial coaggregation

## Abstract

We report the complete genome sequence of the fusobacterial strain PK1594, widely used to study coaggregation between *Fusobacterium nucleatum* and oral bacteria. The assembly comprises a 2,357,192-bp chromosome and a 20,441-bp contig representing a duplicated 10,221-bp plasmid. *znpA* analysis indicates that PK1594 is *Fusobacterium hwasookii* rather than *F. nucleatum*.

## ANNOUNCEMENT

*Fusobacterium nucleatum* is a gram-negative bacterium associated with various oral diseases and systemic infections (1, 2). A defining characteristic of this organism is its ability to aggregate with diverse oral bacterial species (2). During early studies investigating the molecular basis of *F. nucleatum*-mediated coaggregation, the following two strains were commonly used: ATCC 25586 and PK1594 (3, 4). *F. nucleatum* represents a highly diverse group currently divided into four subspecies—*nucleatum* (FNN), *vincentii* (FNV), *polymorphum* (FNP), and *animalis* (FNA). Based on genetic, genomic, and biochemical evidence, ATCC 25586 has been classified as FNN (5, 6). However, no gene sequence or classification data were available for strain PK1594 until now.

Strain PK1594 was originally isolated from a human subgingival site and first reported in 1982 as *Fusobacterium nucleatum* strain VPI E2S-11A (7). In 1989, it was renamed PK1594 when deposited in the laboratory of the late Dr. Paul E. Kolenbrander (NIDCR, NIH) (4). The strain was revived from a –80°C glycerol stock by direct inoculation into 8 mL of trypticase soy broth supplemented with 1% peptone and incubated anaerobically at 37°C for 14 h. The cells were harvested by centrifugation, and the genomic DNA was extracted using the ZymoBIOMICS DNA Miniprep Kit (Zymo Research, D4300) following the manufacturer’s protocol. DNA concentration was determined using a Qubit 4 fluorometer (Thermo Fisher Scientific, Q33327). Sequencing was performed by SeqCenter (Pittsburgh, PA) using both Illumina and Oxford Nanopore Technologies (ONT) platforms.

Illumina libraries were prepared using the Illumina DNA Prep kit, which enzymatically fragments genomic DNA during tagmentation. Libraries underwent bead-based size selection targeting an average insert size of ~280 bp. Paired-end (2 × 151 bp) sequencing was carried out on a NovaSeq X Plus system. All tools were run with default parameters unless otherwise specified. After adapter trimming and quality filtering, Illumina reads totaled 6,888,276 pairs (983 Mbp), with a read N50 of 151 bp and >Q30 quality (≈50× coverage). ONT libraries were prepared using the PCR-free Ligation Sequencing Kit (SQK-NBD114.96) with the NEBNext Companion Module (E7180L), followed by sequencing on a GridION platform with R10.4.1 flow cells (400-bp mode). Base calling was performed with Dorado v0.5.3 (super-accurate model), and FASTQ files were extracted using samtools fastq v1.20 (8). ONT sequencing produced 308,792 reads, totaling ~562 Mbp (N50 = 3,872 bp). Adapter sequences were removed using Porechop. *De novo* hybrid assembly of Illumina and ONT reads was performed with Flye v2.9 (nano-hq model) (9). Assembly quality was assessed using QUAST v5.0.2 (10), which reported a total assembly size of 2,377,633 bp, two contigs, an N50 of 2,357,192 bp, and a GC content of 27.13%. Circularization verified with Circlator v1.5.5 (11), and annotation completed via the NCBI Prokaryotic Genome Annotation Pipeline (PGAP) v5.2 (12). The assembly yielded two circular contigs: a 2,357,192-bp chromosome and a 20,441-bp plasmid contig containing a perfect tandem duplication, indicating a true circular size of ~10,221 bp.

**Fig 1:**
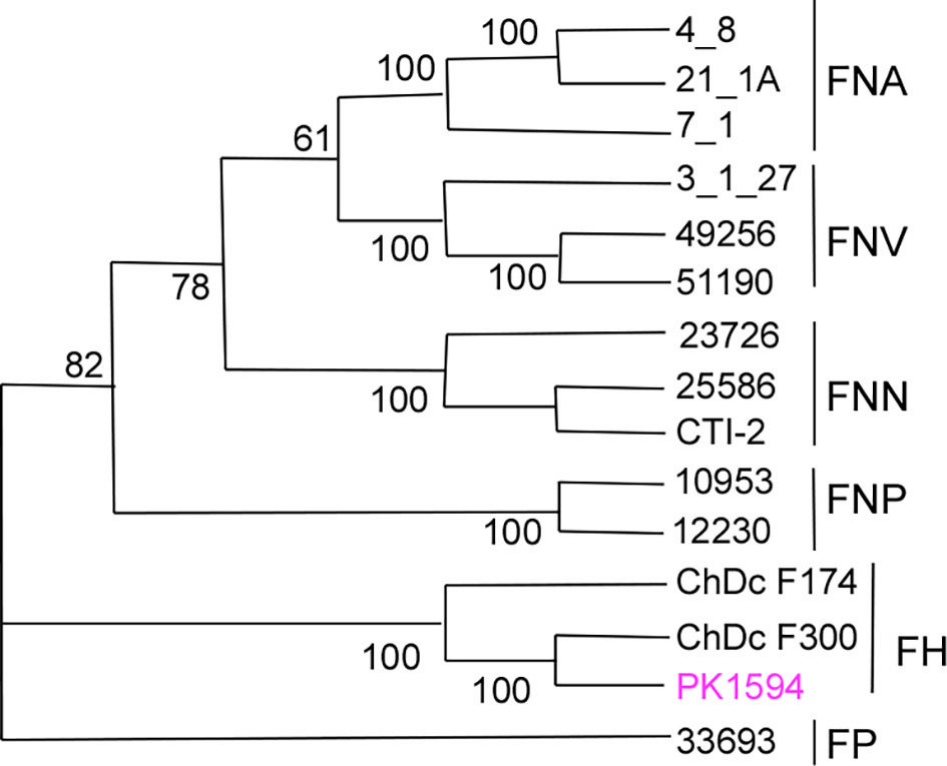
Phylogenetic analysis of strain PK1594 with *znpA* gene. Phylogenetic tree constructed from the full-length zinc protease gene sequence (*znpA*, 1,227 bp) of type strains and clinical isolates of *F. nucleatum*, *F. hwasookii* (FH), and *F. periodonticum* (FP). The tree was generated using the UPGMA method in MEGA-X with the Maximum Composite Likelihood model and 100 bootstrap replicates; bootstrap values are shown at branch nodes. The *zpnA* gene from PK1594 clusters within the *F. hwasookii* clade rather than *F. nucleatum*.

Gene analysis of *znpA* indicated that strain PK1594 belongs to *Fusobacterium hwasookii*, a species that is close to *F. nucleatum* (13) (Fig. 1). The genome encodes homologs of *fap2*, *fadA*, and *fomA* but lacks *radD*. The complete genome sequence of PK1594 provides a foundation for future studies on its physiology, virulence factors, and host interactions.

## Data Availability

The whole-genome sequences of *Fusobacterium hwasookii* strain PK1594 and the endogenous plasmid pPK01 have been deposited in GenBank under the accession numbers JBSGKE010000001 and JBSGKE010000002. Raw Illumina reads are available under SRX31193593, and raw ONT reads are available under SRX31193592. All data sets are associated with BioProject PRJNA1363202 in the Sequence Read Archive (SRA). The *znpA* gene sequences used for phylogenetic tree construction are available at https://doi.org/10.6084/m9.figshare.30611306.v1.
